# Validation of the UNESP-Botucatu unidimensional composite pain scale for assessing postoperative pain in cattle

**DOI:** 10.1186/s12917-014-0200-0

**Published:** 2014-09-06

**Authors:** Flávia Augusta de Oliveira, Stelio Pacca Loureiro Luna, Jackson Barros do Amaral, Karoline Alves Rodrigues, Aline Cristina Sant’Anna, Milena Daolio, Juliana Tabarelli Brondani

**Affiliations:** Department of Anaesthesiology, Faculty of Medicine, UNESP – Univ Estadual Paulista, Botucatu, São Paulo 18618-970 Brazil; Department of Veterinary Surgery and Anaesthesiology, Faculty of Veterinary Medicine and Animal Science, UNESP – Univ Estadual Paulista, Botucatu, São Paulo, 18618-970 Brazil; Institute of Zootechny, Nova Odessa, São Paulo 13460-000 Brazil; Department of Zootechny, FCAV, UNESP – Univ Estadual Paulista, Jaboticabal, São Paulo 14884-900 Brazil

**Keywords:** Cattle, Castration, Orchiectomy, Reliability, Responsiveness, Validity

## Abstract

**Background:**

The recognition and measurement of pain in cattle are important in determining the necessity for and efficacy of analgesic intervention. The aim of this study was to record behaviour and determine the validity and reliability of an instrument to assess acute pain in 40 cattle subjected to orchiectomy after sedation with xylazine and local anaesthesia. The animals were filmed before and after orchiectomy to record behaviour. The pain scale was based on previous studies, on a pilot study and on analysis of the camera footage. Three blinded observers and a local observer assessed the edited films obtained during the preoperative and postoperative periods, before and after rescue analgesia and 24 hours after surgery. Re-evaluation was performed one month after the first analysis. Criterion validity (agreement) and item-total correlation using Spearman's coefficient were employed to refine the scale. Based on factor analysis, a unidimensional scale was adopted.

**Results:**

The internal consistency of the data was excellent after refinement (Cronbach’s α coefficient = 0.866). There was a high correlation (p < 0.001) between the proposed scale and the visual analogue, simple descriptive and numerical rating scales. The construct validity and responsiveness were confirmed by the increase and decrease in pain scores after surgery and rescue analgesia, respectively (p < 0.001). Inter- and intra-observer reliability ranged from moderate to very good. The optimal cut-off point for rescue analgesia was > 4, and analysis of the area under the curve (AUC = 0.963) showed excellent discriminatory ability.

**Conclusion:**

The UNESP-Botucatu unidimensional pain scale for assessing acute postoperative pain in cattle is a valid, reliable and responsive instrument with excellent internal consistency and discriminatory ability. The cut-off point for rescue analgesia provides an additional tool for guiding analgesic therapy.

**Electronic supplementary material:**

The online version of this article (doi:10.1186/s12917-014-0200-0) contains supplementary material, which is available to authorized users.

## Background

The assessment of pain in animals is challenging due to their lack of verbal expression [1]. This challenge is intensified in cattle because, as they are prey in their natural state, they may avoid expressing pain to limit vulnerability [1]. Cattle are routinely subjected to surgical procedures related to management and production, such as dehorning and orchiectomy, usually without adequate analgesia [2-6]. In surveys of veterinarians concerning the use of analgesics in cattle practice, lack of knowledge in recognising pain [6], the belief that farm animals feel less pain than smaller animals [7], economic reasons [8,9] and the lack of valid and reliable instruments to assess pain have been cited as the main reasons why analgesics are not used more frequently [6].

The assessment of pain in cattle is important in determining the need for analgesic intervention, in evaluating the effectiveness of treatment and in comparing the effects of various analgesics. Difficulty in the assessment of pain is not as serious a problem in other domestic species, as there are validated scales in the literature for the assessment of acute pain in dogs [10-13], cats [14-16] and horses [17-20]. In cattle, a scoring system for gait has been validated and found to be reliable and sensitive for identifying cows with severe hoof lesions [21]. However, there are no validated scales for the assessment of acute postoperative pain in cattle.

To develop an accurate tool to assess pain, it is necessary that the scale show validity, responsiveness and reliability [14,22-24]. The primary tool used to assess pain in animals is species-specific behaviour [25]. Although pain signals in ruminants may not be easy to recognise, changes associated with pain can often be seen in the animals’ appearance, posture, gait, appetite, weight, interaction with other animals and with the environment and in the frequency of movement and vocalisation. In addition, animals in pain may protect, lick or bite the wound area [1,8,26-34]. When experiencing pain after orchiectomy, cattle may stomp the ground with their feet, ease their quarters, directing attention to the lesion site, stand still with their limbs extended backward or apart or lie down with their hind limbs extended [27,28]. Such behaviour is absent or reduced when the animal receives adequate analgesia [34].

The scales most often used for measuring postoperative pain are ordinal in nature and can be classified as either unidimensional or multidimensional [35]. Unidimensional scales, such as the simple descriptive (SDS), numerical rating (NRS) and visual analogue (VAS) scales [11,12], only measure pain intensity [36], whereas multidimensional or composite assessment scales also take into account the sensory and affective qualities of pain [13,37].

In developing an instrument to assess pain, it is important to determine the minimum score that indicates a need for analgesic intervention. For this purpose, ROC (Receiver Operating Characteristic) curve analysis may be used. This methodology was first used in veterinary medicine in a pain assessment scale for cats [15,16].

The primary objective of this study was to validate a scale to assess acute pain in cattle subjected to orchiectomy. To that end, the following specific objectives were defined: 1) develop a record of pain-related behaviour; 2) correlate the number of steps taken, time spent lying down and number of lying bouts with the degree of postoperative pain; 3) refine the proposed scale; 4) evaluate inter- and intra-observer reliability, validity and responsiveness; and 5) define a cut-off point for analgesic intervention.

## Methods

The study was approved by the Ethics Committee for Animal Use of FMVZ-UNESP-Botucatu under protocol number 147/2011. Forty Nellore cattle two to three years of age weighing 365 ± 51 kg were used. The animals were considered to be healthy based on clinical and laboratory assessment (complete blood count and biochemical tests).

Prior to the assessment of normal behaviour, the animals were maintained in groups of 3 to 4 for 24 hours in a pasture paddock, with hay and feed placed in troughs and with water *ad libitum*, to permit them to adapt to the new environment. At this point, pedometers (Pedometer Plus®)^a^ were placed on the right forelimbs of the animals to provide a means of measuring the number of steps, time spent lying down and number of lying bouts for 24 hours prior to (baseline - D0) and 24 hours after (D1) surgery [32]. The behaviour of the animals was then filmed intermittently over a 24-hour period to determine the normal behaviour of each animal. Following the 24-hour period of baseline behavioural assessment, an experienced surgeon performed an orchiectomy on each animal using the open technique. Each animal received 0.025 mg/kg xylazine (Anasedan®)^b^ intramuscularly (IM) followed after 10 minutes by the injection of 10 mL of 1% lidocaine without vasoconstrictor (Xylestesin®)^c^ in each spermatic cord and 5 mL in the incision line. Ten minutes thereafter, the orchiectomy was performed. Immediately after xylazine administration, benzathine penicillin (Pentabiótico®)^d^ was administered IM at a dose of 30,000 IU/kg.

After the procedure, the animals were kept in the same enclosure in which they had been housed prior to surgery. Rescue analgesia was applied to all animals after the four-hour assessment at the end of the surgical procedure, with 3 mg/kg ketoprofen (Ketojet®)^e^ and 0.2 mg/kg morphine (Dimorf®)^c^, both administered intravenously (IV) in separate syringes. For the rescue analgesia, the animals were placed in a restraining chute, and the analgesic was administered in the marginal ear vein using a butterfly cannula (19G) after restraint of the head. The analgesic doses were selected based on the literature and on information provided by the manufacturer [31,38].

One observer evaluated and filmed the animals’ behaviour for 25 minutes in each of the following periods: 24 and 16 hours before and 1, 2, 4, 5, 8 and 24 hours after orchiectomy. The animals were filmed using two cameras positioned near the observation paddock. To minimise potential effects of the observer on the animals' behaviour, the observer and cameras were positioned behind a black plastic screen. There were two openings in the screen through which filming and observation could be performed.

The pain scoring scale was developed based on previous studies [27,30,32], on the results of the pilot study and on the analysis of footage taken during the experiment. During the experiment, categories or levels were incorporated, modified or excluded from the original scale. Behaviour was assessed before surgery (M1), at the anticipated time of greatest pain, between 1 and 4 hours after orchiectomy (M2) (according to the pilot study, this was the period during which the animals expressed the most intense pain-related behaviour), one hour after administration of the rescue analgesic (M3) to investigate the efficacy of analgesia and 24 hours after the surgical procedure (M4), totalling 66 hours of video assessment. Based on analysis of the videos obtained at each time period, the percentage duration of specific behaviours was recorded, including time spent eating, ruminating, drinking, walking, standing and in recumbency. Also noted were changes in locomotion and in standing and recumbency posture, interactions with the environment and with other animals and position of the head. This analysis was used to identify behaviours related to pain.

To assess content validity, the scale was sent to three evaluators with expertise in cattle behaviour. These evaluators analysed and scored the behaviour by degree of importance according to the following scale: −1 = irrelevant item; 0 = do not know; 1 = relevant item. The total item correlation was evaluated, and items that achieved a score of ≥ 0.5 were accepted [39].

The proposed instrument yielded a variable score scale composed of behavioural categories. The variables were ordinal in nature and exhibited three descriptive levels, to which a score (a numerical value) was assigned. In the scoring, zero reflected normality and one or two represented changes related to pain, with a maximum score of 16 points.

The same three evaluators who performed the content validity analysis analysed the edited footage to validate the scale. For this purpose, they received a hard drive containing four films, each approximately three minutes long, for each of the 40 cattle evaluated, corresponding to the time points previously described. The chronological order of the videos was randomised so that the evaluators were blinded with respect to the assessed time points, and the descriptions of the animals’ behaviour did not include the pain scores. The researcher responsible for the study, who was considered the local observer, also analysed the videos so that the agreement between the blinded observers and the local observer could be compared to determine inter-observer reliability. One month after the first evaluation, the blinded observers evaluated the videos again with the order of the cattle and videos changed to establish intra-observer reliability.

After watching each film, the observers specified, based on their clinical experience, whether rescue analgesia should be performed and provided sequentially determined pain scores using the Visual Analogue (VAS), Numerical Rating (NRS), Simple Descriptive (SDS) scales and the proposed scale. The data regarding the application of rescue analgesia were used to determine the minimum score related to the need for rescue analgesia.

### Statistical analysis

The Shapiro-Wilk normality test was used to compare the percentage of time during which the animals engaged in specific behaviours (states) during each of the time periods observed in the footage. Because all variables were nonparametric, Friedman's test was used. The paired t-test was used to compare the number of steps taken, the time spent lying down (minutes) and the number of lying bouts during the pre (D0) and postoperative (D1) periods. Differences were considered significant when p < 0.05.

The criterion validity was evaluated based on the agreement between the scores determined by the blinded observers and those determined by the local observer. First, the percentage of absolute agreement for each scale item was determined considering only M2. The percentage of absolute agreement was considered satisfactory when it was ≥ 60%. The weighted kappa coefficient was then calculated with a 95% confidence interval (CI) [40] for each scale item, considering all of the assessment times as a group (MA = M1, M2, M3 and M4). The kappa coefficient results were interpreted according to Altman's classification [41]: 0.81 to 1.0, very good; 0.61 to 0.8, good; 0.41 to 0.6, moderate; 0.21 to 0.4, fair; and < 0.2, poor.

Factor analysis was used to define the number of factors (dimensions or domains) determined by different variables to establish the dimensionality of the scale [42]. Exploratory factor analysis was performed based on principal component analysis, and factors were identified based on the Kaiser criterion, which recommends retaining all components with eigenvalues > 1 [43]. The factor structure was determined by attributing each item with a factor loading and communality > 0.5 to a factor.

The item-total correlation was evaluated using Spearman's non-parametric correlation coefficient between each item and the sum of all scale items. This correlation coefficient was used to evaluate the relevance of each item to the instrument and to identify items that contributed strongly to the total scale score. Items with a correlation coefficient < 0.4 were rejected [42].

The internal consistency of the scale after refinement was evaluated by calculating Cronbach's alpha coefficient [44]. Internal consistency with a value of > 0.70 was considered adequate [45]. The concurrent (criterion) validity was evaluated by comparing the scores obtained using the scale with the scores determined using the VAS, NRS and SDS. Spearman’s correlation coefficient was calculated for each blinded observer and for the local observer as well as for the blinded observers as a group.

To determine the inter-observer reliabilities with or without the local observer and the intra-observer reliability for each scale item, the intraclass correlation coefficient (ICC) with 95% CI was used for MA and for M2 and M4 grouped together [46]. The two-factor model with absolute agreement criterion was selected, and the values obtained were interpreted using Altman's classification, described earlier.

The construct validity was established based on hypothesis-testing methodology. The first hypothesis was that if the scale actually measures pain, the scores after surgery should be higher than the preoperative scores (M1 *versus* M2). The second and third hypotheses were that the scores should decrease after the administration of analgesics and over time (M2 *versus* M3 and M2 *versus* M4, respectively). The scores were expressed as medians, and the Wilcoxon test was used for the analysis of significance (p < 0.05) [16]. This analysis evaluated the responsiveness of the scale.

To determine a minimum score related to the need for intervention or rescue analgesia, ROC (Receiver Operating Characteristic) curve analysis was conducted to provide a graphical representation of the relationship between "true positives" (sensitivity) and "false positives" (1-specificity). The area under the curve (AUC) was also determined; this value indicates the discriminatory ability of the test [47], with AUC values above 0.9 representing high accuracy [48].

## Results

The percentage frequencies of specific behaviours observed in the videos are shown in Table 1. During the time in which they were in greatest pain, the animals spent less time eating and walking and more time exhibiting changes in gait and posture in the standing position or lying down with the head on or close to the ground.

**Table 1:** **Mean values (±SD) of percentage duration of the behaviour of 40 cattle during the perioperative period**

**Behaviour**	**M1**	**M2**	**M3**	**M4**
Eating	55.3 ± 33.4^A^	6.2 ± 14.4^B^	39.0 ± 32.3^A^	34.1 ± 36.8^A^
Ruminating	2.8 ± 19.9	0.0	0.0	5.1 ± 14.3
Drinking	0.4 ± 97.5	0.07 ± 0.42	0.25 ± 0.8	0.1 ± 0.3
Walking	8.8 ± 6.7^A^	0.3 ± 0.7^B^	10.0 ± 17.1^A^	6.7 ± 8.2^A^
Abnormal gait^*^	0.0^B^	4.8 ± 4.8^A^	0.4 ± 1.8^B^	0.5 ± 1.9^B^
Standing/idle	31.7 ± 28.8^B^	25.5 ± 26.7^B^	43.7 ± 32.2^A^	50.5 ± 33.5^A^
Standing/abnormal posture^†^	0.0^B^	16.6 ± 20.7^A^	1.7 ± 11.1^B^	0.0^B^
Lying down	0.0^B^	34.4 ± 24.0^A^	4.7 ± 20.8^B^	1.8 ± 7.6^B^
Abnormal lying down ^‡^	0.0	7.5 ± 15.3	0.0	0.0
Lying down with head resting on/close to the ground	0.0^B^	4.6 ± 6.9^A^	0.0^B^	0.0^B^
Interaction	1.0 ± 1.9	0.07 ± 0.2	0.25 ± 1.2	1.2 ± 4.3
Head below the line of the spinal column	0.0	2.5 ± 6.9	0.05 ± 0.3	0.04 ± 0.3

^A,B^Significant difference between time points (means followed by different letters differ from each other, A > B). *Restricted movement; the line of the spinal column might be normal or hunched and the steps might be shorter when walking. ^†^Rigid and/or caudally extended hind limbs and/or hunched back. ^‡^ Extension of one or more limbs when in ventral recumbency or ventrolateral or lateral recumbency. M1 - preoperative; M2- time of maximum pain; M3 - after rescue analgesia; M4 - 24 hours after the surgical procedure.

Regarding the frequency of the occurrence of specific behaviours, arching the back and extending the neck cranially were observed only at M2 in 4 and 13, respectively, of the 40 animals studied. Kicking, wagging the tail abruptly, looking at and licking the wound were observed more frequently at M2 (21, 7, 14 and 7 of 40 animals, respectively) than at M3 (6, 2, 3 and 0 of 40 animals, respectively) and M4 (7, 0, 1, and 6 of 40 animals, respectively) and were not observed at M1.

No difference between D0 and D1 was observed in the number of steps (D0 – 5401 ± 1142; D1 – 4702 ± 1737; p = 0.08) or in time spent lying down (D0 – 486 ± 265 minutes; D1 – 509 ± 213; p = 0.64); however, the animals lay down more often in the period 24 hours after surgery (D0 – 17 ± 4; D1 – 20 ± 4.9; p = 0.0007).

### Content validity

All of the scale items proposed yielded scores higher than 0.5 regarding the item-total correlation and were thus accepted.

#### Refinement of the proposed scale

##### Percentage of absolute agreement and agreement by the weighted kappa reliability coefficient - criterion validity

The absolute agreement between the scores assigned by the blinded observers and those assigned by the local observer at M2 was considered unsatisfactory for the items *standing posture a*nd *head position* (<60%) based on the analysis of all evaluators. Only one evaluator did not find satisfactory agreement for the items *locomotion*, *interactive behaviour* and *miscellaneous behaviours*, and these items were therefore retained in the scale.

The correlations between each blinded observer’s pain scores and those of the local observer for each item are shown in Table 2. The correlation was fair for the item *standing posture* and ranged from poor to fair for the item *head position*, except in the case of one evaluator, where the agreement was moderate for both items. Based on these results, the items *standing posture* and *head position* were excluded from the scale and from the subsequent analyses.

**Table 2:** **Agreement between the local evaluator and blinded observers for each item on the scale**

**Scale items**	**Blinded observers**		
	**Evaluator 1**	**Evaluator 2**	**Evaluator 3**
Standing posture	0.60 (0.43 – 0.75)	0.26 (0.14 – 0.38)	0.40 (0.26 – 0.54)
Head position	0.51 (0.37 – 0.66)	0.11 (0.03 – 0.19)	0.23 (0.12 – 0.34)
Locomotion	0.56 (0.41 – 0.71)	0.47 (0.34 – 0.61)	0.61 (0.41 – 0.75)
Interactive behaviour	0.65 (0.52 – 0.77)	0.67 (0.58 – 0.75)	0.77 (0.69 – 0.86)
Activity	0.67 (0.55 – 0.79)	0.56 (0.44 – 0.68)	0.74 (0.63 – 0.85)
Appetite	0.86 (0.80 – 0.93)	0.76 (0.67 – 0.86)	0.81 (0.72 – 0.89)
Attention to surgical wound	0.68 (0.55 – 0.82)	0.67 (0.51 – 0.81)	0.85 (0.75 – 0.95)
Miscellaneous behaviours	0.80 (0.72 – 0.81)	0.62 (0.50 – 0.74)	0.84 (0.78 – 0.92)

Agreement between local and blinded observers by weighted kappa coefficient (95% CI) for each item of the acute postoperative pain scale in cattle, covering all evaluation periods (preoperative and postoperative: before and after rescue analgesia and 24 hours after end of surgery). Interpretation: 0.81 – 1.0: very good; 0.61 – 0.80: good; 0.41 – 0.6: moderate; 0.21 – 0.4: fair; <0.2: poor.

##### Factor analysis (construct validity)

After the exclusion of items deemed inappropriate (*standing posture* and *head position*), exploratory factor analysis was conducted on the remaining six items with M2 and M4 grouped together. This analysis generated a factor with an eigenvalue of 3.43. Items other than *attention to the surgical wound* showed satisfactory factor loading and communality (Table 3). The scale was therefore considered unidimensional.

**Table 3:** **Exploratory factor analysis of the acute postoperative pain scale in cattle**

**Scale items**	**Factor load** ^*^	**Communality** ^**#**^
	**Factor 1**	
Locomotion	0.823	0.678
Interactive behaviour	0.860	0.739
Activity	0.863	0.745
Appetite	0.734	0.538
Attention to surgical wound	*0.350*	*0.123*
Miscellaneous behaviour	0.783	0.613
**Eigenvalue**	3.436	
**% Cumulative variance**	57.274	

Exploratory factor analysis based on principal component analysis and with Kaiser criterion (eigenvalue > 1).

*Factor loading represents the correlation between items and factors.

#Communality represents the proportion of variance of each item that can be explained by the factor.

The factor structure was determined considering items with factor loading and communality greater than 0.5.

##### Correlation coefficient of item score with total score

The item-total correlation with M2 and M4 grouped together ranged from 0.395 to 0.848 (Table 4). The item *attention to the surgical wound* was rejected and excluded from the scale because its correlation coefficient was <0.4.

**Table 4:** **Spearman’s correlation coefficient between item score and total score**

**Item**	**r**	**p-value**
Movement	0.836	p = 0.000
Interactive behaviour	0.780	p = 0.000
Activity	0.843	p = 0.000
Appetite	0.848	p = 0.000
Attention to surgical wound	*0.395*	p = 0.000
Miscellaneous behaviour	0.745	p = 0.000

r = Spearman’s correlation coefficient. Interpretation: 0 – 0.35: low correlation; 0.35 – 0.7: moderate correlation; 0.7 – 1.0: high correlation. Correlation coefficient in italic indicates that the item was rejected (r < 0.4).

##### Behaviour included in the miscellaneous behaviour item after refining the acute postoperative pain scale in cattle

Despite the exclusion of three items, one behaviour for each item remained (*standing posture:* hind limbs extended caudally; *head position:* head below the line of the spinal column; *attention to the surgical wound:* licking the wound); these behaviours were included in the item *miscellaneous behaviour* based on the percentage of absolute agreement among the blinded evaluators and the local observer for the grouped time points (MA) and for M2 separately (Table 5).

**Table 5:** **Percentage of absolute agreement between the local and the blinded observers**

**Blinded observers**	**Hind limbs extended caudally**		**Head below the line of spinal column**		**Licks surgical wound**	
	**MA**	**M2**	**MA**	**M2**	**MA**	**M2**
Evaluator 1	91.2%	75.0%	96.2%	87.5%	95.6%	90.0%
Evaluator 2	67.5%	32.5%	93.7%	85.0%	95.6%	92.5%
Evaluator 3	93.1%	85.0%	98.1%	95.0%	97.5%	90.0%

Percentage of absolute agreement for the behaviours *hind limbs extended caudally, head below the line of spinal column* and *licks surgical wound* covering all assessment time points (preoperative and postoperative: before and after rescue analgesia and 24 hours after surgical procedure) and for M2 separately (before rescue analgesia) of the acute postoperative pain scale in cattle.

Degree of satisfactory agreement: ≥ 60.0%.

#### Validation of the UNESP-Botucatu unidimensional pain scale for assessing postoperative pain in cattle

##### Evaluation of the internal consistency of the scale

**Figure 1:**
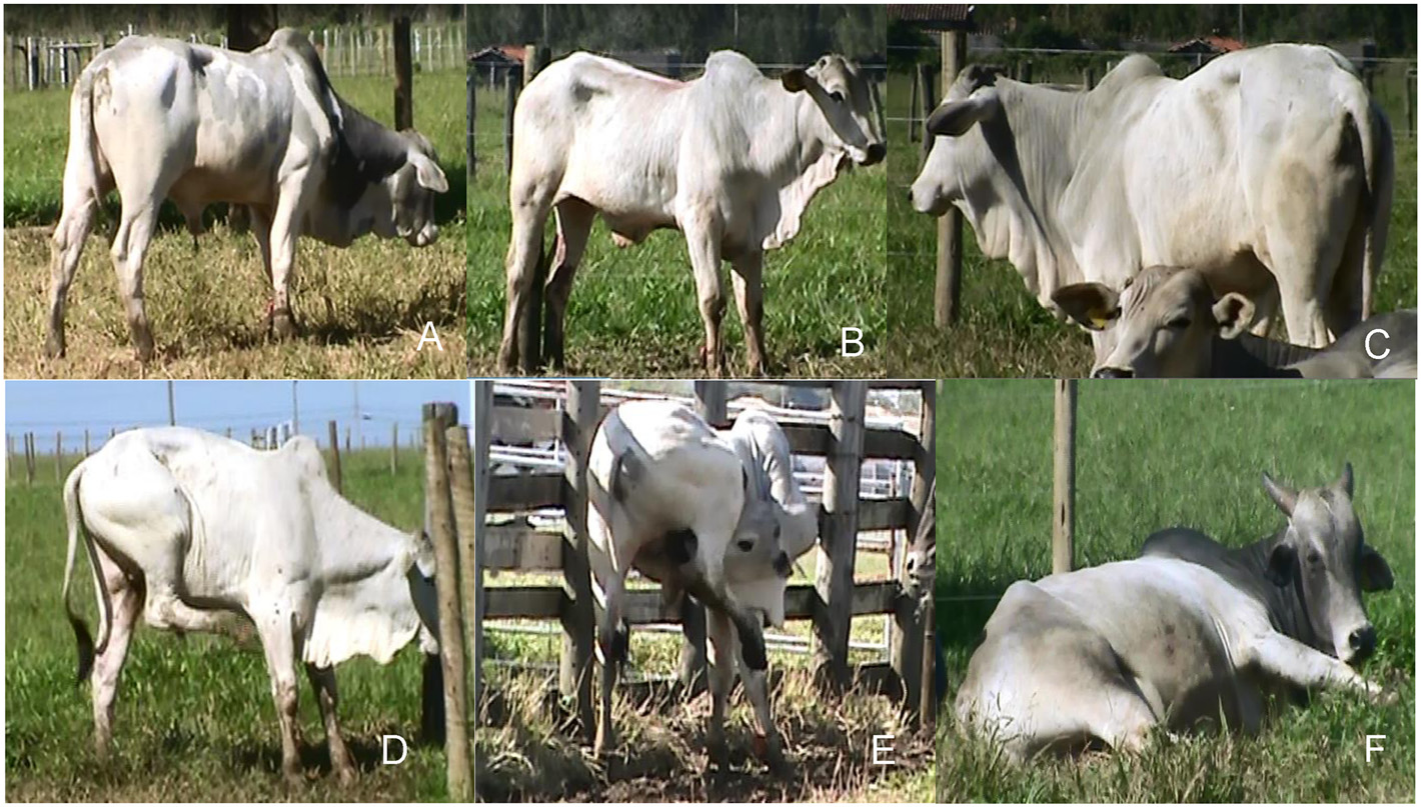
**Characteristic signs of pain in cattle after orchiectomy. A** - Head below the line of spinal column; **B** - Hind limbs extended caudally when in standing posture; **C** - Moves and arches the back when in standing; **D** - Kicking/foot stamping; **E** - Licking the surgical wound; **F** - Lying down in ventral recumbency with full or partial extension of one or both hind limbs.

After refinement, the final version of the UNESP-Botucatu unidimensional pain scale for assessing acute postoperative pain in cattle contained five items, each with three categories (Table 6, Additional file 1: Video 1, Figure 1). The total score was based on the sum of each item, ranging from zero (no pain) to ten (maximum pain).

**Table 6:** **UNESP-Botucatu unidimensional pain scale for acute postoperative pain assessment in cattle**

**Item**	**Score/Criterion**
**Locomotion**	▪ (0) Walking with no obviously abnormal gait.
	▪ (1) Walking with restriction, may be with hunched back and/or short steps.
	▪ (2) Reluctant to stand up, standing up with difficulty or not walking.
**Interactive behaviour**	▪ (0) Active; attention to tactile and/or visual and/or audible environmental stimuli; when near other animals, can interact with and/or accompany the group.
	▪ (1) Apathetic: may remain close to other animals, but interacts little when stimulated.
	▪ (2) Apathetic: may be isolated or may not accompany the other animals; does not react to tactile, visual and/or audible environmental stimuli.
**Activity**	▪ (0) Moves normally.
	▪ (1) Restless, moves more than normal or lies down and stands up with frequency.
	▪ (2) Moves less frequently in the pasture or only when stimulated.
**Appetite**	▪ (0) Normorexia and/or rumination.
	▪ (1) Hyporexia.
	▪ (2) Anorexia.
**Miscellaneous behaviours**	▪ Wagging the tail abruptly and repeatedly.
	▪ Licking the surgical wound.
	▪ Moves and arches the back when in standing posture.
	▪ Kicking/foot stamping.
	▪ Hind limbs extended caudally when in standing posture.
	▪ Head below the line of spinal column.
	▪ Lying down in ventral recumbency with full or partial extension of one or both hind limbs.
	▪ Lying down with the head on/close to the ground.
	▪ Extends the neck and body forward when lying in ventral recumbency.
	(0) All of the above described behaviours are absent.
	(1) Presence of 1 of the behaviours described above.
	(2) Presence of 2 or more of the behaviours described above.

The Cronbach's α coefficient of the scale after refinement was 0.866, indicating that the instrument has excellent internal consistency and lending weight to the feasibility of using the total score to interpret the results obtained.

##### Concurrent validity (criterion validation)

**Figure 2:**
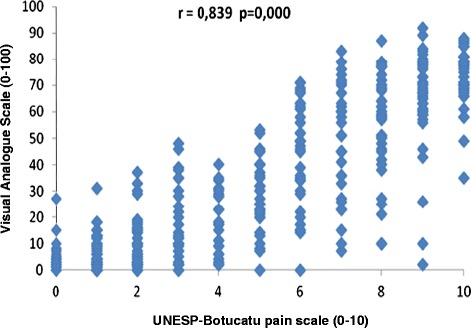
**Correlation between pain scores recorded using the UNESP-Botucatu unidimensional pain scale and the VAS.**

**Figure 3:**
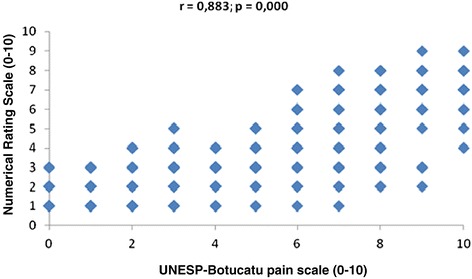
**Correlation between pain scores recorded using the UNESP-Botucatu unidimensional pain scale and the NRS.**

**Figure 4:**
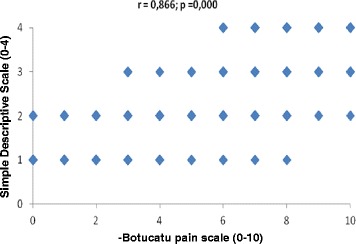
**Correlation between pain scores recorded using the UNESP-Botucatu unidimensional pain scale and the SDS.**

When considering MA, a high correlation was observed between the pain scores determined using the UNESP-Botucatu unidimensional pain scale and the scores determined using the VAS (r = 0.839), NRS (r = 0.883) and SDS (r = 0.866), taking into account all blinded evaluators (Table 7, Figures 2, 3 and 4).

**Table 7:** **Correlation between the UNESP-Botucatu unidimensional pain scale and the VAS, NRS and SDS**

**Scales**	**Local observer**	**Blinded observers**			**Total**
		**1**	**2**	**3**	
VAS	0.812*	0.847*	0.884*	0.860*	0.839*
NRS	0.869*	0.835*	0.895*	0.912*	0.883*
SDS	0.823*	0.854*	0.874*	0.910*	0.866*

*p = 0.000. Interpretation of Spearman’s correlation coefficient: 0 – 0.35: low correlation; 0.35 – 0.7: moderate correlation; 0.7 – 1.0: high correlation.

##### Inter-observer reliability

The agreement of observers considering both MA and M2 and M4 as a group ranged from moderate to good (Table 8).

**Table 8:** **Agreement between blinded observers, including or not including the local observer, for each scale item**

**Scale items**	**Blinded observers**		**Local and blinded observers**	
	**MA**	**M2 and M4**	**MA**	**M2 and M4**
Locomotion	0,50 (0,34 – 0,62)	0,44 (0,23 – 0,60)	0,52 (0,42 – 0,62)	0,45 (0,31 – 0,59)
Interactive behaviour	0,68 (0,56 – 0,75)	0,63 (0,50 – 0,74)	0,69 (0,62 – 0,75)	0,63 (0,52 – 0,73)
Activity	0,57 (0,45 – 0,67)	0,54 (0,38 – 0,67)	0,61 (0,52 – 0,69)	0,56 (0,44 – 0,67)
Appetite	0,80 (0,72 – 0,85)	0,76 (0,91 – 0,84)	0,80 (0,75 – 0,85)	0,77 (0,68 – 0,84)
Miscellaneous behaviours	0,66 (0,58 – 0,73)	0,72 (0,63 – 0,80)	0,71 (0,65 – 0,76)	0,75 (0,68 – 0,82)

Agreement between blinded observers, including or not including the local observer, for all grouped assessment time points (preoperative and postoperative: before and after rescue analgesia and 24 hours after end of surgery) and for M2 and M4 grouped together (postoperative, after rescue analgesia and 24 hours after surgery) for the UNESP-Botucatu unidimensional pain scale for assessing acute postoperative pain in cattle using the intraclass correlation coefficient (95% CI). Interpretation: 0.81 – 1.0: very good; 0.61 – 0.80: good; 0.41 – 0.6: moderate; 0.21 – 0.4: fair; <0.2: poor.

##### Intra-observer reliability

For MA, the intra-observer reliability ranged from good to excellent (Table 9). When considering M2 and M4 as a group, the reliability for evaluator 1 ranged from good to very good, whereas the reliability for evaluator 2 ranged from moderate to good. For evaluator 3, the reliability was good.

**Table 9:** **Intra-observer reliability for each scale item and observer**

**Scale items**	**Blinded observers**		
	**Evaluator 1**	**Evaluator 2**	**Evaluator 3**
Locomotion	0.67 (0.57 – 0.75)	0.62 (0.52 – 0.71)	0.69 (0.60 – 0.77)
Interactive behaviour	0.81 (0.75 – 0.86)	0.74 (0.66 – 0.80)	0.77 (0.69 – 0.82)
Activity	0.80 (0.74 – 0.85)	0.63 (0.52 – 0.71)	0.69 (0.60 – 0.77)
Appetite	0.96 (0.95 – 0.97)	0.82 (0.77 – 0.87)	0.81 (0.75 – 0.85)
Miscellaneous behaviours	0.82 (0.77 – 0.87)	0.61 (0.50 – 0.70)	0.78 (0.71 – 0.83)

Agreement between observers at all grouped assessment time points (preoperative and postoperative: before and after rescue analgesia and 24 hours after surgery) using the intraclass correlation coefficient (CI 95%). Interpretation: 0.81 – 1.0: very good; 0.61 – 0.80: good; 0.41 – 0.6: moderate; 0.21 – 0.4: fair; <0.2: poor.

##### Construct validity

The construct validity was determined according to the changes in pain scores in response to the surgical procedure (M1 *versus* M2), after administration of analgesics (M2 *versus* M3) and throughout the postoperative period (M2 *versus* M4). The total scale score increased significantly postoperatively (M2) compared to M1 (Table 10) and decreased significantly after the administration of analgesics (M3) and throughout the postoperative period (M4) compared to M2, thereby indicating construct validity. Based on these data, it can also be stated that the scale shows responsiveness.

**Table 10:** **Total scores determined by local and blinded observers**

**Time points**		**Blinded observers**		
	**Local observer**	**1**	**2**	**3**
M1	0 (0–2)	0 (0–3)	0 (0–6)	0 (0–2)
M2	7 (3–10)*	7.5 (0–10)*	8 (0–10)*	7 (1–10)*
M3	0 (0–8)^†^	1 (0–9)^†^	4 (1–10)^†^	0.5 (0–8)^†^
M4	1 (0–5) ^†^	0 (0–7) ^†^	3.5 (1–8) ^†^	0 (0–6) ^†^

Medians and minimum and maximum values of total score for the UNESP-Botucatu unidimensional pain scale (0 – 10) determined by local and blinded observers based on videos obtained during the perioperative period of cattle subjected to orchiectomy.

* Pain scores at M2 significantly higher than at M1 (p < 0.001).

† Pain scores at M3 and M4 significantly lower than at M2 (p < 0.001).

##### Determination of the cut-off point - ROC curve

**Figure 5:**
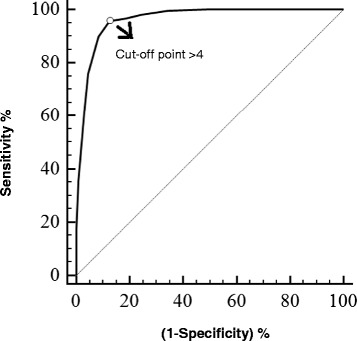
**ROC curve and optimum cut-off point > 4 for rescue analgesia.** ROC (*Receiver Operating Characteristic*) curve for the UNESP-Botucatu unidimensional pain scale demonstrating an optimum cut-off point > 4 for rescue analgesia with a sensitivity of 95.8%, a specificity of 87.3% and an area under the curve of 0.963.

**Figure 6:**
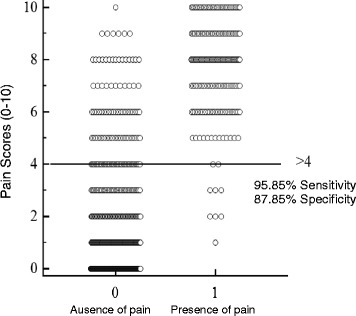
**Diagram illustrating the optimum cut-off point identified from the ROC curve analysis.**

Different cut-off points were suggested by analysis of the ROC curve. When the point simultaneously representing the highest sensitivity and the highest specificity was identified (Table 11), an optimum cut-off of > 4 (scale range 0–10) with a sensitivity of 95.85% (95% CI: 92.3 to 98.1%) and a specificity of 87.35% (95% CI: 84.7 to 89.7%) was established (Figures 5 and 6). Additionally, the high AUC observed, 0.963 (95% CI: 0.949 to 0.974, p < 0.0001), indicates that the instrument has excellent discriminatory ability.

**Table 11:** **Determination of optimum cut-off based on ROC curve analysis**

**Cut-off point**	**Sensitivity (95% CI)**	**Specificity (95% CI)**
≥ 0	100.00 (98.3 – 100.0)	0.00 (0.0 – 0.5)
> 0	100.00 (98.3 – 100.0)	51.14 (47.5 – 54.8)
> 1	99.54 (97.5 – 100.0)	65.55 (62.0 – 69.0)
> 2	98.16 (95.3 – 99.5)	75.24 (72.0 – 78.3)
> 3	96.77 (93.5 – 98.7)	81.29 (78.3 – 84.0)
> *4*	*95.85 (92.3 – 98.1)*	*87.35 (84.7 – 89.7)*
> 5	89.86 (85.1 – 93.5)	91.66 (89.4 – 93.5)
> 6	76.04 (69.8 – 81.6)	95.56 (93.8 – 96.9)
> 7	61.29 (54.5 – 67.8)	97.04 (95.6 – 98.1)
> 8	35.48 (29.1 – 42.2)	99.06 (98.1 – 99.6)
> 9	17.51 (12.7 – 23.2)	99.87 (99.3 – 100.0)
> 10	0.00 (0.0 – 1.7)	100.00 (99.5 – 100.0)

The determination takes into consideration the blinded observers’ assessments regarding the need or lack of need for analgesia.

## Discussion

Because animals are unable to report their pain as humans can [1], the recognition of pain in animals requires the ability to understand the behaviour of the target species, the behavioural changes typically observed in animals in pain and the specific changes that occur in each animal’s behaviour in response to pain. In this context, the video records obtained in this study served as an initial survey of items that might be appropriate for use in building and subsequently validating a scale for the assessment of pain in cattle.

The use of video recording for the validation of scales and for behavioural assessment is a common tool [14-16,20] that permits the simultaneous analysis of an animal by multiple evaluators to be performed as often as necessary. In this study, following evaluation of the videos by the researcher, the films were edited according to the behaviour observed at different points in time. After reviewing the films, changes not covered in the initial scale were identified and items deemed irrelevant were excluded. The behaviour of animals in pain (M2) showed a reduction in eating and moving around, and when animals in pain did move around, they did so with restrictions and/or short steps and/or hunched backs. In addition, animals in pain spent more time lying down with their heads on or near the ground. When in the standing position, these animals assumed an abnormal posture, e.g., hunched and rigid and/or with the hind limbs extended caudally. Arched-back movements were also observed more frequently in animals in pain, along with cranial extensions of the neck while lying down, kicking, wagging the tail abruptly and looking at and licking the surgical wound. Given their relationship to pain, these behaviours were incorporated into the scale.

Some of the behaviours observed in this study have been described previously in cattle subjected to orchiectomy. These behaviours include remaining idle for longer periods, assuming an abnormal standing posture [26,49] and exhibiting gait changes involving shorter, more cautious steps [32,33]. With respect to time spent lying down, the results reported in the literature vary according to whether xylazine and analgesics were used. It would seem that animals spend more time in the standing position when xylazine is not used [26,30,31,50] and that they spend more time lying down and less time moving around when it is used [33,51]. In the present study, although xylazine might contribute to lying down after the surgery, the fact that administration of additional analgesia resulted in less rather than more lying behaviour suggests that this behaviour is due to pain rather than to the sedative effect of xylazine.

The use of xylazine in the present study was necessary because the Nellore breed is skittish and difficult to control. No animal showed recumbency after orchiectomy when leaving the restraining chute, once again demonstrating that the sedation was mild. Low doses of xylazine (0.015 to 0.025 mg/kg IV or IM) generally promote sedation without recumbency in ruminants [38]. In conclusion, decreased activity in cattle may be a good indicator of pain.

The reduced time spent eating observed in our study is consistent with other findings in the literature that report reductions in grazing time [29,50], eating frequency [49,50,52] and, in the case of calves, suckling time [26]. The benefit of rescue analgesia with respect to this behaviour was also evident from the fact that the time spent feeding increased after rescue analgesia was performed.

Kicking and abrupt wagging of the tail were observed more frequently at M2, as described previously [26,27,29]. These events may occur after the local anaesthetic effect has lost its effectiveness [29] but may also be related to the presence of flies, which might represent one limitation of the study. Although it is impossible to completely eliminate flies from the environment, care was taken to reduce the number of flies present by using fly repellent, and the study was conducted in the winter when there is a low incidence of insects. One possible indication that flies had little effect on the kicking and abrupt tail wagging observed in this study was that kicking the abdomen was not observed at baseline; furthermore, the behaviour of wagging the tail abruptly and repeatedly is very characteristic and differs from the motion the animal makes to ward off flies. Thus, it would appear that these two behaviours are also related to pain in cattle [26].

Although it might be expected that the number of steps taken by the animals would be reduced after orchiectomy, no significant difference was observed in the number of steps recorded before and after orchiectomy. This finding differs from results previously reported in the literature [32]. The difference may be explained by the fact that the animals in the previous study did not receive analgesia [32], in contrast to this study, in which there was only a short (4-hour) span during which analgesia was not provided. The short period of pain experienced by the animals in the present study was most likely insufficient to influence the data obtained during the 24-hour evaluation.

The larger number of lying bouts observed after orchiectomy may be related to the restlessness and discomfort of the animals in the period prior to the application of the rescue analgesic. A similar phenomenon was observed in previous studies in cattle [27,31]. Lying-down behaviour is also evaluated on the pain scales commonly used for dogs [10,12,13] and cats [14,16], showing that although it is important to develop species-specific tools to assess pain, some pain behaviours are common among species.

Because the pedometer is not expensive and is relatively easy to handle, it can be a useful tool in the assessment of pain in cattle, especially when data analysis is carried out over relatively short periods.

Methods other than pain scales have also been used to investigate pain following castration in cattle. These methods include the assessment of physiological and neuroendocrine changes, such as serum cortisol concentration, and infrared thermography [53]. Facial expression of pain and kinematic and force platform gait analysis have been used in mice and in horses and dogs, respectively [54-56]. However, these methods have not yet been validated in cattle, and they either do not provide information in real time or require special equipment that is not currently available and/or is impractical under field conditions.

Validity and reliability are the key attributes of a scale that can be used to identify and quantify pain in animals. Reliability demonstrates the ability of the scale to reproduce the results regardless of the evaluator and at different times by the same evaluator [22]. In this study, the assessment of content validity was performed using the judgment of experts in the field who analysed the representativeness of each item in relation to the scale as a whole [57]. This methodology, which is well accepted [16,17,42], refers to the scope and adequacy with which the instrument reflects the phenomenon of interest, in this case, pain [22].

Criterion validity tests the effectiveness of a scale's measurement by comparing results obtained using that scale to results obtained using a previously validated method [12]. Criterion validity can be predictive when evaluating the criterion after testing and concurrent when evaluating the instrument and the criterion simultaneously [57]. In tests of criterion validity, the correlation between the scale and another instrument, ideally the gold standard [16,22], is evaluated.

Considering that, to our knowledge, no gold standard instrument has been developed to evaluate pain in cattle and that correlation of the total scores obtained using our proposed scale with the scores determined by VAS may be questionable, an alternative method was used to investigate criterion validity in this study. The method involved comparing the agreement between pain scores assigned by blinded evaluators and a “gold standard” evaluator, in this case, the local evaluator. This method has been used with instruments designed for use in cats [16] and in young children [58].

Although the VAS, SDS and NRS may not show inter-observer reliability when tested on animals, they are nonetheless widely used to validate veterinary pain scales [12,13,16,59] because the gold standards of verbal expression and self-assessment evaluation are not available in animals. Although inter-observer reliability may not be adequate when using VAS [59], intra-observer agreement or reliability is consistent over time [37] and may be a good option for measuring and comparing pain assessed by the same trained observer over time, as was done in this study [37].

The same methodology used for criterion validity in studies in cats [14,15] was used to refine the scale by comparing the pain scores determined by blinded observers with those determined by the local evaluator. Subsequently, criterion validity should be evaluated by correlating the results obtained using the proposed scale and another instrument considered the gold standard (concurrent validity) [13]. Given the absence in the literature of validated scales for pain assessment in cattle, the pain scores on the scale proposed in this study were compared with the scores obtained using three other classical scales used in animals, the VAS, the NRS and the SDS. There was a high correlation between the results obtained using the four scales. Although these scales have not been validated in animals, this approach has been widely used to evaluate pain scales in veterinary medicine [12,13,16,20].

Using factor analysis, it is possible to determine the dimensionality of the scale [45], i.e., the number of factors (dimensions or domains) represented by different variables [42]. Because the scale in question generated only one factor, it was considered unidimensional, in contrast to the scales validated for cats, which were considered multidimensional based on this analysis [16,42]. Factor analysis is commonly used to develop an instrument and to relate a large number of variables such that the items that define specific parts of the construct are grouped together [60].

Despite the low reliability observed for the items *standing posture* and *head position* and the low correlation of the item *attention to the surgical wound* with the total scale score in this study, it was deemed important to retain a behaviour for each item.

The behaviours *rigid hind limbs*, *hunched back* and *head below the line of spinal column* may not have been clearly visible in the videos, and this may have resulted in the observed poor correlation between the blinded observers and the local observer on the items *standing posture* and *head position*. Conversely, the behaviour *head below the line of spinal column* obtained satisfactory agreement when considering only M2. Regarding the item *attention to the surgical wound*, the description of the behaviour *looking at the surgical wound* may not have been wholly appropriate because it produced different results when assessed by the local evaluator and the blinded observers. A description such as *moves the snout in the direction of the surgical wound* might have clarified observation of this behaviour; the description *looking at the surgical wound* was subjective because it could denote looking at the abdomen and/or to the side for another reason, a fact that may have confused the observers.

According to the Cronbach's α value, the scale employed in this work has excellent internal consistency [45,60]. Internal consistency ensures that the scores of the items comprising the scale can be summed to produce a total score related to the overall assessment of pain intensity [16].

The moderate to good inter-observer agreement found in this work demonstrates the consistency of the results obtained by different evaluators and the ability of the instrument to produce consistent results [23]. The lower level of agreement for the item *locomotion* may be due to the short video analysis time and the animals’ way of walking, which may have hindered the definition of the category. Thus, the results of both inter- and intra-observer reliability tests demonstrated good repeatability and stability of the scale.

The analysis grouping of M2 and M4 was important to confirm the reliability of the scale because these points represent the two most challenging times for pain assessment. A similar approach was used to validate a postoperative acute pain scale in cats [15,16], but in that case, only M2 was considered separately. In our study, M4 was included because it also represents a challenging time, given the reduction in analgesic effect and the manifestation of pain-related behaviour that typically occurs after 24 hours.

Construct validity examines whether a given instrument detects predictable changes in the construct [22]. It can be evaluated by the well-known group method. This method determines whether the instrument detects differences between groups and is based on testing the hypothesis that time and intervention, both surgical and analgesic, should alter the pain scores [16]. The observed differences between pain scores at the time of greatest pain (M2) and the scores at other time points confirm the construct validity used in this work by verifying the reduction in pain scores in response to analgesia and over time [36]. This method has also been used to validate scales in veterinary medicine [14,16] and attests to the responsiveness of the scale using a similar approach.

ROC curve analysis was used to determine the minimum score required for analgesic intervention [48], as was previously performed for a pain assessment scale in cats [15,16]. The determination of scores that suggest a need for the use of analgesics assists the professional’s clinical decision, affirms the effectiveness of analgesic treatment [15] and helps avoid unnecessary suffering in animals. Based on the balanced sensitivity and specificity criteria observed in this study, an optimum cut-off of > 4 was identified, i.e., additional analgesia is recommended when the pain score is ≥ 5 (0–10 point scale). It should be emphasised that according to clinical evaluation, additional analgesia must be performed if deemed necessary even if the score is lower than the cut-off point.

The high AUC observed (0.963) in this study indicates that the scale has excellent discriminatory ability and high accuracy, i.e., the instrument can correctly classify subjects with or without pain [47,48]. Similar results were observed in the validation of a pain scale in cats [15,16].

A possible limitation of this study is the absence of a control or uncastrated group of animals. The inclusion of a control group was considered when the study was designed, and a pilot study was performed to address this point. Subsequently, the authors decided to use only one castrated group and a larger number of animals based on the rationale that the animals’ behaviour during the time period immediately prior to surgery could be considered a control because, at this point, the animals had already adapted to the environment and no management changes were performed during the study that could influence the results. This methodology has previously been used in cats [14,16], dogs [12,13] and horses [20]. The results observed here, which show significant changes in pain scores before surgery, after surgery and after analgesia, support the validity of the construct as well as the responsiveness of the scale. Additional support for the idea that the pre-surgical time period provided an appropriate control comes from the fact that the observers were blinded to the test moments and the order of the videos was randomised to avoid any bias. Although, in a very few cases, it was possible to observe the region of the testicles in the videos, it was not possible to determine from the video footage whether the animal had already been castrated. Another consideration is that it would be difficult to compare a different, uncastrated control group of animals with a group of castrated animals because the response to pain varies according to each individual.

The results of this study allow us to state that the UNESP-Botucatu unidimensional pain scale for assessing acute postoperative pain is valid and reliable. However, clinical tests with different analgesics and surgical protocols are recommended to assess the scale’s clinical applicability.

## Conclusions

It is concluded that, following the refinement of the originally proposed scale, the UNESP-Botucatu unidimensional pain scale for assessing acute postoperative pain in cattle is a valid, reliable and responsive instrument with excellent internal consistency and discriminatory ability. The cut-off point for rescue analgesia provides an additional tool for guiding analgesic therapy.

## Endnotes

^a^SAE Afimilk, Israel.

^b^Vetbrands, Paulínia, São Paulo, Brazil.

^c^Cristália, Itapira, São Paulo, Brazil.

^d^Fort Dodge, Campinas, São Paulo, SP.

^e^Agener, Embu Guaçu, São Paulo, SP.

## Additional file

Additional file 1: Video 1. Normal behaviours and pain behaviours in cattle.
